# The immediate effect of a single session of pain neuroscience education on pain and the autonomic nervous system in subjects with persistent pain, a pilot study

**DOI:** 10.7717/peerj.11543

**Published:** 2021-05-31

**Authors:** Rob Sillevis, Gabriel Trincado, Eric Shamus

**Affiliations:** Rehabilitations Sciences, Florida Gulf Coast University, Ft. Myers, FL, United States of America

**Keywords:** Neuro pain science education, Autonomic nervous system, Pupillometry, Chronic pain

## Abstract

**Background:**

The autonomic nervous system is a system that operates at the subconscious level and has been associated with neurobehavioral aspects of pain. Overall, persistent pain has a stimulating effect on the sympathetic nervous system. A promising emerging nonpharmacological treatment to manage persistent pain is neuroscience-based pain education. The overarching goal of neuroscience-based pain education is to change cognitions about pain and the pain experience through education. The aim was to determine the immediate and short-term impact of a neuroscience-based pain education video on the autonomic nervous system and pain in a subgroup of individuals with persistent pain.

**Methods:**

A convenience sample of 26 subjects were recruited for this study. Each subject indicated their pain level at the time of testing using a Visual Analogue Scale. Automated pupillometry was utilized to measure pupil diameter. After two minutes of accommodation to the goggles, the pupil was measured continuously for 60 s. Following this a 5-minute video presentation “Understanding Pain” was watched, followed by a continuous pupil measurement for 60 s. Three minutes after this measure, the final pupil diameter measurement was taken for 60 s. After completing the final pupil measure, the subject was asked to fill out a second Visual Analogue Scale and a Global Rate of Change.

**Outcomes:**

Each subject completed a Global Rating of Change Scale and the mean score was 1.14 (SD = 1.61 and a SEM = 0.), supporting the hypothesis of an overall self-perceived benefit from the intervention. There was a statistically significant difference in pain following the video, *P* < 0.01. A significant correlation was observed between the self-perceived decrease in pain level and the Global Rating of Change score, *p* = 0.02. There was no statistically significant difference in the mean pupil diameter following the video with *p* = 0.76 for the right eye and *p* = 0.250 for the left eye.

**Discussion:**

This pilot study demonstrated that a 5-minute neuroscience-based pain education video reduced perceived pain in a small sample of subjects with persistent pain. Watching the neuroscience-based pain education video did not seem to result in an immediate generalized autonomic nervous system response. However, it resulted in a different reaction on each eye. This unequal response might be the result of the hemispheric lateralization of the ANS. This study supports the fact that the pain experience is determined by the balance between conscious cognitive processes and subconscious processes based on previous psychological experiences.

## Introduction

Persistent pain is a global societal issue with a 40% prevalence (Boswell et al., 2005). The International Association for the Study of Pain identifies that the presence of pain past the normal stages of healing and longer than three months should be considered persistent pain (Raja et al., 2020). Persistent pain results in a significant societal burden with increased health care costs, the inability to work, and loss of work-related productivity (Gross et al., 2004; Gross et al., 2002; Tseng et al., 2006). Persistent pain has been defined as “an unpleasant sensory and emotional experience associated with, or resembling that associated with, actual or potential tissue damage” (Raja et al., 2020), Many different disorders/conditions can result in persistent pain; for example, chronic spinal pain has been reported to be as high as 80% (Abdi et al., 2007; Boswell et al., 2005; Manchikanti et al., 2002). Up to 60% of patients with acute cervical related pain will continue to have symptoms five years after the initial onset of symptoms (Boswell et al., 2005; Manchikanti et al., 2008; Manchikanti et al., 2007). The National Institute of Health indicates that persistent pain is the leading cause of long-term disability in the US (Raja et al., 2020).

Historically, the management of persistent pain has been challenging (Shadbolt et al., 2020). Accurate management of persistent pain conditions requires reliable and valid pain assessment and identifying pathophysiological mechanisms underlying the pain. To achieve this, it is essential to understand that pain has many different domains, including the sensory and affective qualities of pain, the temporal dimension of pain, and the location and bodily distribution of pain (Fillingim et al., 2016). The integration of both conscious and subconscious processes within the human body will lead to pain awareness, which partially depends on previous experiences. Reliable and valid pain assessment is the basis for effective intervention.

Currently, self-report measures are the gold standard of pain assessment (Dworkin et al., 2005). However, these methods have some shortcomings. Self-report measures which solely rely on conscious, cognitive, and rational processes and may not, therefore, be the most suitable tools to capture one’s true pain experience fully (Verastegui-Tena et al., 2017). Pain is the direct result of the interpretation of nociceptive stimulation in the brain (Jackson et al., 2020). Initially, peripheral nociceptors, which are present in great abundance throughout all tissues, are activated as a result of actual tissue damage (Bernards & Bouman, 1988; Coutaux et al., 2005; Cyriax, 1982; Raja et al., 2020). Damaged tissues produce chemical mediators such as serotonin, ATP, glutamate, and cytokines. These chemical mediators lower the facilitation threshold of nociceptors causing peripheral sensitization (Amaya et al., 2013) in a state of peripheral sensitization continuous nociceptive information will reach the spinal cord. As a result of ongoing nociceptive input the excitation threshold of the inter-neurons will lower (Fernandez-de-Las-Penas et al., 2020; Staud et al., 2001). When this threshold is lower, normal sub-threshold afferent signals will now result in central discharge resulting in a state of central sensitization (Fernandez-de Las-Penas et al., 2020; Fong & Schug, 2014; Woolf & Doubell, 1994). Both peripheral and central sensitization have been correlated with a change in autonomic nervous system activity (Matsuda, Huh & Ji, 2019).

The autonomic nervous system (ANS) operates at the subconscious level and has been associated with neurobehavioral aspects of pain (Cotton et al., 2018). It has been identified that dysfunction of the ANS could contribute to the development of painful conditions (Di Franco et al., 2009; Hallman & Lyskov, 2012). It has also been demonstrated that stress-related dysregulation as a result of pain results in changes in ANS activity (Di Franco et al., 2009). Overall, persistent pain has a stimulating effect on the sympathetic nervous system (Kawasaki, 1999; Loewenfeld & Newsome, 1971; Yang, Niemann & Larson, 2003). Because the ANS is involuntary, it is less susceptible to personal bias and therefore, might provide insight into the individual’s pain experience. Previously physiological variables such as sweat responses, blood pressure and heart rate, and local tissue circulation were used to identify autonomic functioning (Butler, 2000; Menck, Requejo & Kulig, 2000). More recently, pupillometry has been used to obtain a real-time impression of autonomic functioning (Sillevis & Cleland, 2011; Sillevis et al., 2019). The ANS exclusively innervates the muscles controlling the pupil diameter, and thus the pupil diameter serves as a direct reflection of ANS activity (Bertinotti et al., 2002; Bitsios, Prettyman & Szabadi, 1996; Capao Filipe et al., 2003; Fotiou et al., 2000). Therefore, dilation of the pupil is the result of a relatively unopposed increase in sympathetic nervous system activity (Bernards & Bouman, 1988; Butler, 2000; Gibbons, Gosling & Holmes, 2000). Fully automated pupillometry has been used previously to capture the pupil diameter in real-time effectively (Sillevis & Cleland, 2011; Sillevis et al., 2010; Sillevis et al., 2019).

A promising emerging nonpharmacological treatment to manage persistent pain is neuroscience-based pain education (Louw et al., 2019a; Louw et al., 2016). Pain Neuroscience Education (PNE) is a biopsychosocial education tool to explain to patients the concepts of sensitization, cortical reorganization, the brain’s role in pain, spinal inhibition, environmental factors, and psychological perception of pain (Goudman et al., 2019; Talmage, Wilmarth & Guffey, 2020). PNE uses metaphors and stories to explain and rationalize the pain experience (Gallagher, McAuley & Moseley, 2013; Louw et al., 2011; Louw et al., 2019b; Moseley, Nicholas & Hodges, 2004). The delivery of the educational material is a significant factor to consider when determining its effects. Written forms of PNE have shown little benefit (Van Ittersum et al., 2014). Face-to-face delivery methods have shown the most favorable outcomes in pain perception and catastrophizing behaviors (Louw et al., 2011). Video-based PNE is a more modern form of communication and can contain different modes of communication in its delivery; however, studies on the clinical relevance of video-based PNE are lacking (Heathcote et al., 2019). The overarching goal of PNE is to change cognitions about pain and the pain experience through education. There might be a dual benefit of PNE for persistent pain patients. The first benefit is a down-regulating of the negative experience of pain by reducing distress-related responses. The reduced stress-related response will directly affect the functioning of the amygdala, hypothalamic-pituitary-adrenal axis, and autonomic nervous system (Tabibnia, 2020). A second possible effect is up-regulating the positive experience of PNE, which in turn can buffer the effects of stress (Tabibnia, 2020). Pain neuroscience education has been shown to decrease pain, reduce fear avoidance, and increase patient knowledge of the pain neurophysiology in patients with a variety of musculoskeletal pain conditions (Goudman et al., 2019; Louw, Puentedura & Mintken, 2012; Louw et al., 2016; Rufa, Beissner & Dolphin, 2019 Talmage, Wilmarth & Guffey, 2020).

No previous studies have reported the direct effect of PNE on the ANS. Therefore, the first aim was to determine the immediate and short-term impact of a PNE video on the autonomic nervous system and pain in a subgroup of individuals with persistent pain. It was hypothesized that awareness about pain mechanisms would lead to a measurable reduction in sympathetic nervous system activity. This secondary aim was to investigate if the PNE video resulted in a reduction of self-perceived pain, a significant change on the Global Rating of Change (GROC) score and if these changes correlate with a change in ANS activity.

## Material and Methods

### Subjects

This pilot study used a method of convenience sampling with a within-subject repeated measure design. Twenty-six subjects were recruited, and all available subjects were screened for eligibility criteria. Inclusion criteria included subjects aged 18 to 65 years and able to read and understand the English language, so proper written consent could be given and the subject could understand the PNE video. The subjects had to have a previously diagnosed persistent pain condition lasting more than three months and experience pain at the day of testing. Exclusion criteria included any diagnosed autonomic disease, central nervous system damage, retinal disease, or infectious causes of pain as they would impact the ANS normal functioning. This study received institutional review board (IRB) approval (# 2019-40) from Florida Gulf Coast University. Written consent was obtained from all subjects before participating in the study.

### Automated measures

**Figure 1:**
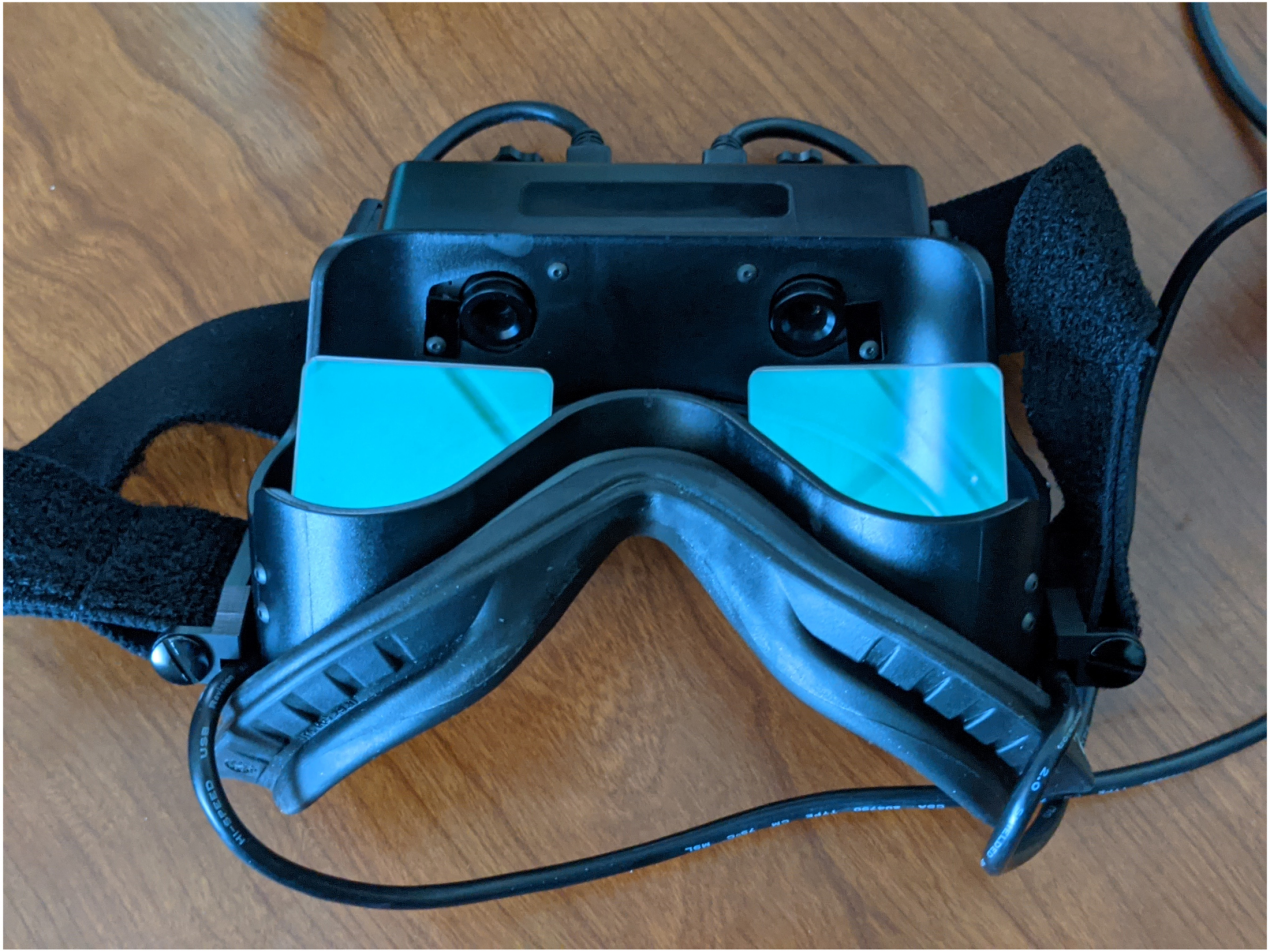
Goggles with infra red camera. The goggles include an infrared camera for each eyes so the pupil diameter of each each is measured.

The pupil diameter was measured directly using the fully automated Vorteq^®^ system (Micromedical Technologies, Inc). This fully automated system uses goggles with two built in infrared cameras to simultaneously measure the pupil of both eyes (Fig. 1). The measurement protocol was based on similar studies using fully automated pupillometry to determine the short-term treatment effect on the ANS (Sillevis & Cleland, 2011; Sillevis et al., 2010; Sillevis et al., 2019). Infrared cameras allow for a precise measurement of the pupil resulting in minimal measurement error. Automated measures allows the identification of changes in pupil diameter of less than 0.2 mm (Meeker et al., 2005; Pop, Payette & Santoriello, 2002; Taylor et al., 2003; Twa et al., 2004). Because the Vorteq system is fully automated and automatically captures the pupil diameter, it has high face validity and strong inter- and intra-rater reliability (Boxer Walcher & Krueger, 1999; Boxer Walcher & Krueger, 2000). Both the sensitivity and reliability of the automated pupillometry to evaluate the autonomic nervous system have been shown previously (Bertinotti et al., 2002; Giakoumaki et al., 2005; Miciele et al., 1995; Neil & Smith, 1989).

### Pain Neuroscience Education (PNE) video

To provide each participant with the same pain neuroscience education experience, an electronic teaching method was used. A 5-minute video presentation “Understanding Pain” with concepts of pain neuroscience education was selected because it addresses serval different domains of pain, including information on pain and the brain, plasticity of the nervous system, differences between chronic and acute pain, sensitization, psychosocial components affecting pain, the use of images and examples of the topics (Louw et al., 2011; Moseley, Nicholas & Hodges, 2004). The video was created by the Defense & Veterans Center for Integrative Pain Management (DVCIPM) the Joint Pain Education Program’s support as part of an initiative to educate patients on common pain management. The Understanding Pain video is freely available for the public at http://www.youtube.com/watch?v=C_3phB93rvI&t=77s.

### Study protocol

After providing written consent all subjects completed a brief self-response survey, which collected data on the subject’s recent caffeine, prescription, and nonprescription medication usage. Each subject indicated their pain level at the time of testing using a Visual Analogue Scale (VAS). The VAS consists of a 100-millimeter line with an anchor at each end. The left anchor indicated “no pain”, and the right anchor indicated “the worst pain imaginable” (Bird & Dickson, 2001; Ries, 2005). The VAS is a common valid and reliable method of subjective assessment of pain in subjects with persistent pain (Bijur, Silver & Gallagher, 2001; Gallagher, Liebman & Bijur, 2001; Jensen, 2003; Jensen, Connie & Brugger, 2002; Jensen et al., 1999; Ries, 2005).

**Figure 2:**
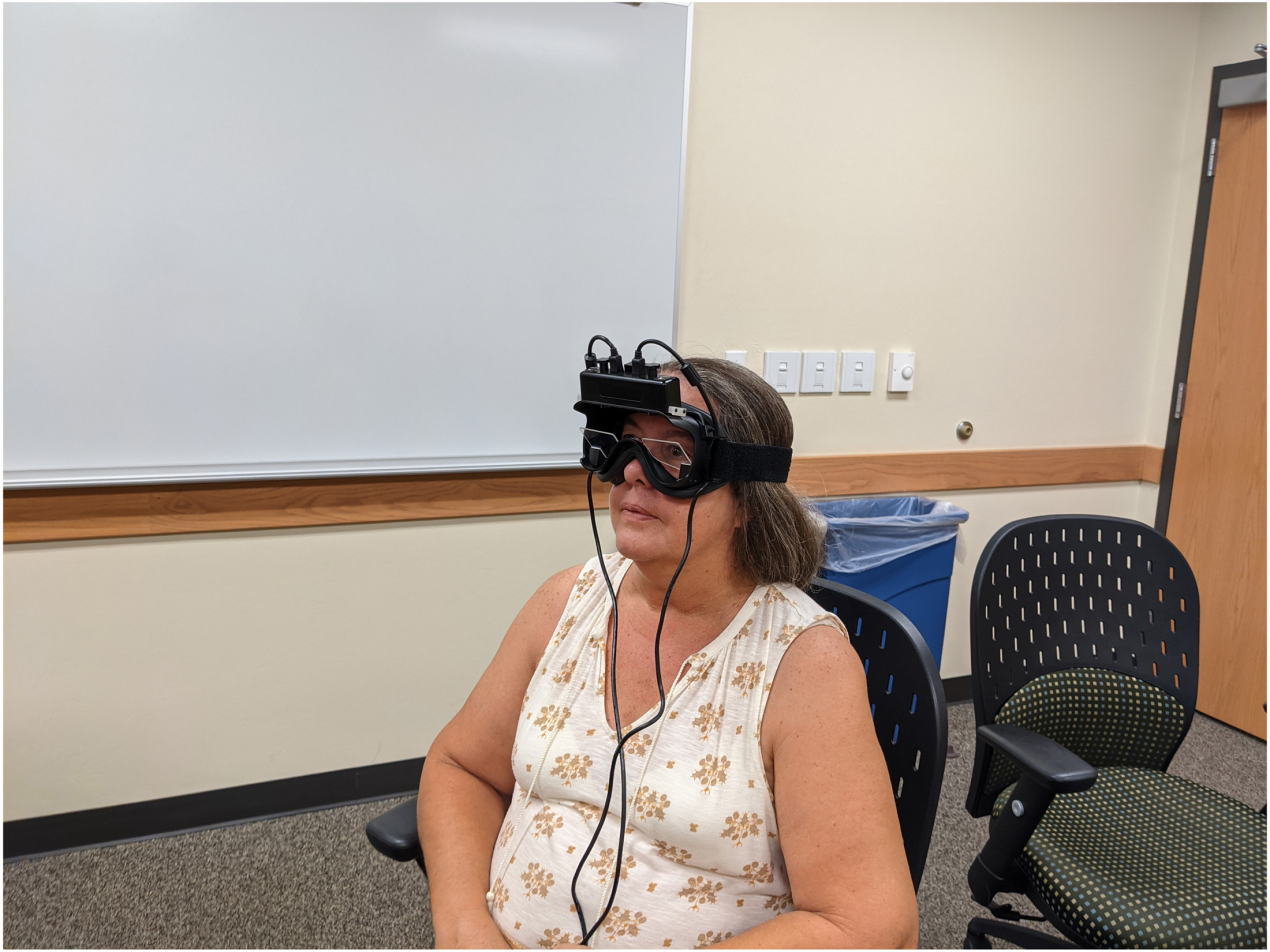
Subjects with goggles. Subject seated in front of TV screen with goggles placed so pupils are captured by the infra red cameras in the goggles.

The testing environment was temperature-controlled and during the measurement phase, all subjects were seated in front of a table on which a monitor was placed to view the PNE video. After being seated, the goggles were placed so that the cameras caught the pupils of both eyes (Fig. 2). After two minutes of accommodation to the goggles, the pupil diameter of both eyes was measured continuously for a 60-second duration. Directly following the baseline measurement, the subject viewed the 5-minute PNE “Understanding Pain” video. Immediately following the video, a continuous pupil measurement of both eyes was performed for 60 s. Three minutes after this measure, the last and final pupil diameter measurement was taken for a duration of 60 s. After completing the final pupil measure, the goggles were removed, and the subject was asked to fill out a second VAS and a Global Rate of Change (GROC) scale (−5 to +5) to determine if they felt any change between how they felt before and after the PNE video. The subjects each had the opportunity to record any additional comments regarding their experience of their participation in the trial on the same form.

### Statistical analysis

Statistical analyses were performed using SPSS, version 25.0, statistical software package from IBM. All data were analyzed using a confidence interval of 95% and a significance level of 0.05. The data were analyzed for normal distribution using the Sharpo-Wilk test of normality. VAS reported data were normally distributed with *P* > 0.05; for that reason, the assumption for parametric statistics was satisfied. The pupil diameter data of both eyes were not normally distributed with *P* < 0.05, for that reason the assumption for parametric statistics was not satisfied. To determine the effect of the PNE video on self-perceived pain levels, the paired-sample *t* test was used. The Pearson correlation was used to determine the correlation between a change in self-perceived pain levels and the Global Rating of Change Scale. To determine the effect of the PNE video on the pupil of both the right and left eye, the Wilcoxon Signed Rank test was used. To assess if there was a correlation between a change in self-perceived pain and pupil diameter change, the Spearman Rank Correlation test was used.

## Results

### Baseline characteristics

A total of 26 subjects were assessed for eligibility. All subjects met the inclusion criteria and were enrolled in the study. All subjects completed the study protocol. Three subjects were male, while twenty-three subjects were female. The mean subject age was 50.73 years with a range of 21 to 65 years. The average pain at baseline was 38.3 mm. The average pupil diameter of the left eye was 82.51, and the right pupil diameter was 67.95 (Table 1).

**Table 1:** Baseline characteristics. Pain self report on Visual Analog Scale in milimeters and pupil diameter measured in pixels.

Descriptive statistics						
	N	Minimum	Maximum	Mean		Std. Deviation
				Statistic	Std. Error	
Age	26	21	65	50.73	2.63	13.42
L average pupil size	26	35.01	210.18	82.51	9.17	46.73
R average pupil size	26	33.97	160.29	67.95	6.55	33.38
pre pain lv 1–10	26	6 mm	83 mm	38.3 mm	38 mm	19.5 mm
Valid N (listwise)	26					

### Global rating of change scale

Each subject completed a Global Rating of Change Scale using a 5-point Likert scale (−5 indicating “very much worse”, 0 indicating “no change”, and 5 indicating “completely gone”) (Sillevis, Shamus & Filho, 2020). Two subjects reported a worsening of the condition. Eight subjects reported feeling unchanged after watching the video. Seven subjects reported a “1” score of change. Four subjects reported a “2” score of change (25.9%). Two subjects reported a “3” score of change. One subject reported a “3.5” score, and two subjects reported a “4.0” score. The mean score was 1.14 (SD = 1.61 and a SEM = 0), supporting the hypothesis of an overall self-perceived benefit from the intervention on their experience.

### The immediate effect of PNE video on pain levels

To determine the immediate effect of Pain Neuroscience Education video on self-perceived pain level, the VAS measure before watching the PNE video was compared to the pain measurement taken immediately after watching the video. The assumptions for the use of parametric statistics were satisfied for the pain measures. Thus, a paired *t*-test test was used to compare the pre-and post-pain scores. The results showed a statistically significant difference in mean VAS values between both measures following the intervention; *t*(26) =7.739, *p* < 0.01. Hence, it was concluded that the PNE “Understanding Pain” video resulted in an immediate pain reduction in our subject pool.

### Correlation between change in pain and global rating of change

A Pearson’s Correlation was used to determine if there was a correlation between the change in self-perceived pain levels and the subject’s perception of change in condition measured on the GROC. A significant correlation was observed between the self-perceived decrease in pain level and the GROC; *p* = 0.02. The correlation was moderately strong between the two measures with *r* =−0.587. This indicates that a reduction in pain correlated inversely with a positive GROC score.

### Immediate effects of NPE video on pupil diameter

The Friedman’s ANOVA was used to measure the difference in pupil diameter from pre video pupil diameter to the post one and post two measures. Table 2 indicates the difference in the mean pupil diameter (measured in pixels) at each of the measurement points for both eyes. When comparing the right eye’s pupil diameter, we observed no statistically significant difference in the mean pupil diameter *p* = 0.76. When comparing the pupil diameter of the left eye, no statistically significant difference was observed in the mean pupil diameter *p* = 0.250. Thus, it was concluded that although the right eye demonstrated dilation of the pupil and the left eye showed a constriction of the pupil overall, it does not seem that the PNE video had an immediate effect on the functioning of the ANS.

**Table 2:** Global rating of change scale. Measured on Likert scale with −5 indicating “very much worse”, 0 indicating “no change”, and 5 indicating “Completely gone”.

		Frequency	Percentage
Valid	−3.0	1	4.0
	−1.0	1	4.0
	.0	8	28.0
	1.0	7	28.0
	2.0	4	16.0
	3.0	2	8.0
	3.5	1	4.0
	4.0	2	8.0
	Total	26	100.0

### Correlation between change in pain and pupil diameter

The Spearman rank correlation was used to determine if there is a correlation between the immediate change in the pupil diameter of both eyes and the change in self-reported pain. When comparing the difference in pupil diameter in the left eye after watching the PNE video to the change in pain, a minimal correlation was observed between the measures with *rs* = −0.283 that was not significant; *p* = 0.162. When comparing the difference in pupil diameter in the right eye after watching the PNE video to the change in pain, no correlation was observed between the measures with *rs* = −0.023 that was not significant; *p* = 0.913. Thus, it was concluded that there is no significant correlation between the self-perceived change in pain and the pupil diameter in either eye.

## Discussion

The aim of the study was to determine the immediate and short-term effect of a PNE video on pain and the autonomic nervous system in a subgroup of individuals with persistent pain. It was hypothesized that awareness about pain mechanisms would lead to a measurable reduction in sympathetic nervous system activity. The study’s secondary aim was to investigate if the PNE video resulted in a reduction of self-perceived pain, a significant change on the Global Rating of Change (GROC) score and if these changes correlated with a change in nervous system activity.

Persistent pain can involve both local and central mechanisms. At the local level, chemical mediators such as serotonin, bradykinin, and potassium are effective stimulants for nociceptors lowering their facilitation threshold (Bernards & Bouman, 1988; Chen & Levine, 2005; Curatolo, Arendt-Nielsen & Petersen-Felix, 2004). It appears that these mediators, which can be generated in local tissues, can cause a prolonged hypersensitivity of peripheral nerves resulting in peripheral (bottom–up) sensitization of the nervous system (Curatolo, Arendt-Nielsen & Petersen-Felix, 2004; Kingery et al., 2000; Sluka et al., 2003). Additionally, hyperactivity can take place at the level of the spinal cord as a result of sensitization (top–down) of the central nervous system (Nijs, Van Houdenhove & Oostendorp, 2010). Sensitization as a result of bottom-up and top-down processes will result in maintaining the persistent pain cycle, and this will have an effect on the sympathetic nervous system (Kawasaki, 1999; Loewenfeld & Newsome, 1971; Yang, Niemann & Larson, 2003). Typically, pain causes an increase in sympathetic nervous system activity and or an inhibiting the parasympathetic nervous system (Kawasaki, 1999; Loewenfeld & Newsome, 1971; Oka et al., 2007; Yang, Niemann & Larson, 2003). During a state of central sensitization, the human body will take those actions to limit painful movements (Zusman, 2002). Such activity can impact functional abilities and treatment outcomes negatively. The ANS is involuntary and, therefore, less susceptible to personal bias (Butler, 2000; Menck, Requejo & Kulig, 2000; Vicenzino, Collins & Wright, 1994). The pupil is innervated exclusively by the autonomic nervous system. Therefore, the pupil diameter can be considered the real-time balance between the sympathetic and parasympathetic nervous systems (Bernards & Bouman, 1988; Elenkov et al., 2000; Filipe et al., 2003; Gibbons, Gosling & Holmes, 2000; Maguire et al., 2007). In this study, pupil responses after watching a PNE video were captured with a fully automated pupillometry method. Fully automated pupillometry has been validated and shown to be reliable when measuring autonomic nervous system activity and avoid examiner bias (Bertinotti et al., 2002; Bitsios, Prettyman & Szabadi, 1996; Boev et al., 2005; Boxer Walcher & Krueger, 1999; Boxer Walcher & Krueger, 2000; Meeker et al., 2005; Merritt, Keegan & Mercer, 1994; Pfeifer et al., 1982; Taylor et al., 2003; Twa et al., 2004).

Immediately after viewing the PNE video, the subjects in this study had a difference in pupil diameter between both eyes in which the mean of the left eye was larger than the right eye. This difference in right-left pupil diameter appears common and has been previously reported (Poynter, 2017). The mean left eye pupil diameter demonstrated a reduction in diameter following the PNE video, which would indicate an immediate reduction in sympathetic activity. This is in contrast to what happened to the mean right eye pupil in our subjects. The mean pupil diameter increased immediately after the PNE video and decreased in width at the final measure; however, overall it remained larger than the baseline measure. This increase in pupil diameter seems to indicate that there was a relative increase in sympathetic activity. It could be possible that the PNE video resulted in a response that demonstrates lateralization of the functioning of the ANS instead of a generalized ANS response based on video content and the different areas of the brain processing this. This finding concurs with a similar finding by Poynter (2017), who demonstrated that side to side pupil diameter symmetry was linked to psychological factors, brain asymmetry, and autonomic arousal which could have influenced a more one sided ANS response in our subjects watching the PNE video. Further studies should investigate if the ANS is able to have brain dominant responses specifically.

This study confirms previous findings that PNE can result in an immediate change in pain perception (Goudman et al., 2019; Louw et al., 2019a; Louw et al., 2016; Talmage, Wilmarth & Guffey, 2020). The mean change in pain levels in our subjects was 11 mm on the VAS after watching the 5-minute video. Alghadir et al. (2018) report the minimally detectable change on the VAS for chronic pain is eight mm. The mean change in pain met this criterion; however, the minimal clinically important difference for the VAS in subjects with chronic pain of 13 mm was not met (Alghadir et al., 2018). Not only did the subjects in this pilot study report a significant immediate reduction of pain, but they also reported an overall change in condition as indicated by the GROC findings. The mean change on the GROC was 1.14, which meets the minimally detectable change on the GROC of .45 (Kamper, Maher & Mackay, 2009). The minimal clinically important difference for the GROC has been reported as 2 points (Kamper, Maher & Mackay, 2009). Seven subjects (27%) met this criterion. This video was the only PNE intervention that was provided, and the relatively short duration of the video could have had an impact on the overall effectiveness of this NPE session. This study’s finding is in contrast with previous reports that PNE as a stand-alone intervention is not beneficial (Louw et al., 2011). Additionally, most previous studies had more extended sessions of PNE, which included human interaction allowing for discussion and in detail explanations of PNE topic matters. PNE is typically used in combination with other interventions; thus, the true effectiveness of PNE delivered by video, the duration, and the frequency and topics of video instruction as a stand-alone intervention should be further investigated (Louw et al., 2011; Verastegui-Tena et al., 2017).

The results of this study support the position that there are both conscious and subconscious processes within the human body that result in the pain experience. It has been demonstrated that environmental and psychological factors throughout one’s life might affect autonomic reactivity to pain (Cotton et al., 2018). This might indicate that conscious awareness and learning might change subconscious functioning over time (Azam et al., 2016). The PNE appeared to have a direct cognitive effect on the pain experience. However, the ANS activity did not change significantly, implying that it might take more for the subconscious systems to change overall functioning than a single intervention. It could have been possible that the ANS in a non-threatening situation will take longer to display a generalized response. Additionally, the subjects in this study all had persistent pain. They likely were in a central sensitized state and despite the reduction in pain still reported pain, and therefore, subconsciously could have remained sympathetic dominant overall. It was only measured until 5 min after the intervention which might have been a limiting factor and could have resulted in missing a generalized ANS change if there was one.

This pilot study has several limitations. First, the small subject sample could have led to a type II error and thus limits the generalizability of the findings. Secondly, there was an unequal gender representation, as the subject sample included three males and 23 females. Gender differences in pupil response to pain have been reported previously (Ellermeier & Westphal, 1995). Therefore, it is possible this could have had a negative effect on the outcomes of this study. This concurs with the findings of Poynter (2017), who identify that males have a greater hemispheric lateralization, which could lead to right-left pupil differences. Future studies should evaluate the possible lateralization of autonomic activity and its effect on target organs such as the pupil or heart. This pilot study measured pupil diameter in a controlled environment, and other than the PNE video, no intervention occurred. All subjects were seated, and it is possible that this position contributed to pain awareness. The pupil diameter was only measured immediately after watching the PNE video and, therefore, long-term effects cannot be inferred. Future studies should measure long-term effect on the ANS. Another limitation was that there was no control for the type of video that was watched. It could have been possible that watching any type of video, PNE or non-PNE, could have an impact on the nervous system. Future studies should evaluate the possible lateralization of autonomic activity and its effect on target organs such as the pupil or heart based on the type of video watched.

## Conclusion

This pilot study demonstrated that a 5-minute PNE video reduced perceived pain in a small sample of subjects with persistent pain. Watching the PNE video did not seem to result in an immediate generalized autonomic nervous system response. However, it resulted in a different reaction on each eye. This unequal response might be the result of the hemispheric lateralization of the ANS. This study supports the fact that the pain experience is determined by the balance between conscious cognitive processes and subconscious processes that are based on previous psychological experiences. Future studies are needed to determine if PNE can be stand-alone intervention and in what format this would be most beneficial to manage subjects with persistent pain.

##  Supplemental Information

10.7717/peerj.11543/supp-1 Supplemental Information 1 Group age and pupillometry raw data

## Additional Information and Declarations

### Data Availability

The following information was supplied regarding data availability: Raw data are available as a Supplementary File.
